# Non-sustained ventricular tachycardias, conduction disorders and an impaired left ventricular ejection fraction in a 32-year-old woman

**DOI:** 10.1007/s12471-019-01326-8

**Published:** 2019-08-28

**Authors:** S. Alsters, Y. Polyukhovych, H. Bikker, L. Wong, A. C. Houweling

**Affiliations:** 1 Department of Clinical Genetics, Amsterdam UMC, Vrije Universiteit Amsterdam, Amsterdam, The Netherlands; 2 Department of Cardiology, Amsterdam UMC, Vrije Universiteit Amsterdam, Amsterdam, The Netherlands; 3 Department of Clinical Genetics, Amsterdam UMC, University of Amsterdam, Amsterdam, The Netherlands

**Fig. 1:**
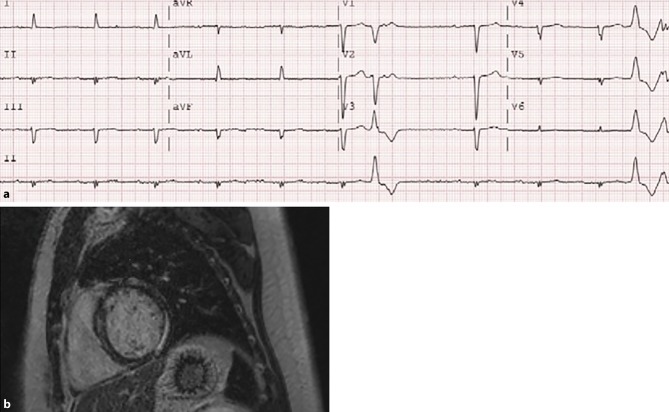
**a** Electrocardiogram. **b** Cardiac MRI

A 32-year-old woman was referred for cardiological evaluation due to palpitations. She did not have a history of syncope. Her family history was negative for sudden death. Electrocardiography showed a sinus rhythm with a first-degree atrioventricular (AV) block (PR interval 310 ms) and premature ventricular contractions (Fig. 1a). A monomorphic non-sustained ventricular tachycardia was seen on Holter recording. Cardiac MRI showed an impaired left ventricular ejection fraction (LVEF) of 41% with mid-myocardial late enhancement consistent with cardiomyopathy (Fig. 1b). DNA analysis revealed a previously reported pathogenic mutation, c.1130G>A p.(Arg377His) in the *LMNA* gene. The cardiac phenotype associated with mutations in the *LMNA* gene typically includes early-onset AV conduction disorders, tachyarrhythmias, dilated cardiomyopathy, in some cases associated with skeletal myopathy [1, 2]. The presence of non-sustained ventricular tachycardias, LVEF <45% at first evaluation, male sex and non-missense mutations (e.g. ins-del/truncating or mutations affecting splicing) are associated with an increased risk of malignant ventricular arrhythmias in *LMNA* mutation carriers [3].
